# *Fusarium concolor* X4 Pretreatment Suppresses Light-Induced Yellowing of High-Yield Pulp

**DOI:** 10.1155/2020/9497215

**Published:** 2020-02-01

**Authors:** Daolei Zhang, Xuezhi Li, Jian Zhao

**Affiliations:** ^1^State Key Laboratory of Microbial Technology, Shandong University, Qingdao 266237, China; ^2^Department of Bioengineering, Shandong Polytechnic, Jinan 250104, China

## Abstract

High-yield pulps (HYPs), such as CTMP (chemi-thermo-mechanical pulp), are attractive due to their low cost and high wood utilization. However, their drawback of rapid brightness reversion (yellowing) limits wide use of the HYPs. In this study, a fungus, *Fusarium concolor* X4, was applied to treat poplar CTMP for exploring the effects of biotreatment on brightness and light-induced yellowing of the pulp. The results indicated that the biotreatment with *Fusarium concolor* X4 could improve the brightness of poplar CTMP and inhibit light-induced yellowing of the pulp. The yellowing inhibition mechanism was explored by the analysis of enzyme production regularity during biotreatment, changes in chemical components, and the UV-Vis absorption spectra and FTIR-ATR spectra of pulps before and after biotreatment.

## 1. Introduction

The increasing forest resource consumption and environmental pollution are the two most serious problems for the pulp and paper industry. Researchers have done intensive studies for these problems [1]. Efficient utilization of forest resources in paper industry is important for both industry and society [2, 3]. Compared with chemical pulps such as kraft pulp, HYPs, with their high utilization efficiency of wood, are a dramatic way to resolve the problems of wood shortages and higher capital costs. In addition, HYPs can also give fine papers with higher stiffness, bulk, and opacity and better printability [4].

However, the well-known “brightness reversion (also called yellowing)” of HYPs is a major drawback in HYPs application [5]. Generally, pulp yellowing includes light- and heat-induced yellowing. For HYPs, because of the high lignin content, light is the main cause for yellowing. The lignin-based reactions, which include the phenoxyl pathway, the ketyl pathway, the phenacyl pathway, and the phenoxyl quinone redox cycle [6, 7], could produce quinones and some other UV-active substances, leading to pulp brightness reversion. Besides, the hexenuronic acid (HexA), low molecular organic compounds, and metal ions in pulp could also cause pulp brightness reversion [8].

According to previous researches, there are many methods to suppress the pulp yellowing. For example, UV absorbers are efficient substances to prevent brightness reversion. It had been reported that thiols or sodium hypophosphite were effective for preventing the light-induced yellowing [9, 10]. 1-oxyl-2,2,6,6-tetramethylpiperidin-4-ol, an inhibitor, was found that could help maintain paper brightness for at least one year [11]. Fluorescent whitening agents (FWAs), chemical modification, and paper coating are also effective methods that can be applied to slow down pulp yellowing. Although these methods could retard yellowing of HYPs, high cost and toxicity of some chemicals limit their industrial application; on the other hand, they will only temporarily delay the yellowing [5].

In recent years, biotreatment is increasingly applied for pulping and bleaching processes in pulp industry because of its environmental friendliness and low cost. Many researchers had done lots of works in this field [12–14]. For example, *E. grandis* wood was treated with fungus *C. subvermispora* to produce bleached bioTMP (thermomechanical pulp) and found the bleached biotreated pulp had better thermal-stability than the control, while the photo-stability was similar for both pulps [15]. Microorganisms especially white rot funguses are rich in enzymes for degradation of lignin and hemicellulose, such as xylanase, laccase, MnP, LiP, etc. [16, 17]. These enzymes can degrade chromophores of pulp and its precursors, which are well beneficial to inhibit pulp yellowing [15, 18, 19]. Besides, the biotreatment could make pulps easier to be bleached and refined, thus decrease the consumption of bleaching agents and save considerable electrical energy, and the pulp strengths of burst, tear, and tensile were also improved [18].

*Fusarium concolor* X4 (*F. concolor* X4) is a fungus that has efficient delignification ability, which was screened and preserved in our laboratory. Our previous studies showed treatment with *F. concolor* X4 could effectively improve brightness and inhibit heat-induced yellowing of unbleached poplar CTMP and wheat straw semichemical pulp [20–22]. In this paper, we studied the effect of *F. concolor* X4 pretreatment on light-induced yellowing of poplar CTMP, and the mechanism of yellowing inhibition was studied by investigating the changes on different types of enzymes produced by the strain and on chemical components of pulps, the UV-Vis absorption spectra, and FTIR-ATR spectra before and after the biotreatment.

## 2. Materials and Methods

### 2.1. Pulps

Unbleached Poplar CTMP was kindly provided by Hua tai Paper Co., Ltd. (Shandong, China). The pulp was washed twice with tap water and preserved at 4°C.

### 2.2. Microorganism

*F. concolor* X4 was screened from decayed wood and maintained on PDA slants in our laboratory [20].

### 2.3. Precultivation

The media (80 mL) contained 20 g/L glucose, and 5 g/L yeast extracted powder was used for precultivation of *F. concolor* X4. The strain was grown for about 3 days at 28°C at 150 rpm to inoculate the shake flask until the cell concentration reached 10^8^ Colony-Forming Units per 1 mL (CFU/mL).

### 2.4. Biotreatment of Pulp with *F. concolor* X4 and Sampling

Poplar CTMP (40 g oven dry weight) were adjusted to 5% consistency with solution (NH_4_Cl 0.2 g, MgSO_4_·7H_2_O 0.5 g, KH_2_PO_4_ 0.2 g, Na_2_HPO_4_ 0.2 g, FeSO_4_·7H_2_O 0.007 g, MnSO_4_ 0.035 g, CuSO_4_·5H_2_O 0.007 g dissolved in 1 L of distilled water) in flasks (3 L) and autoclaved at 115°C for 30 min. After cooling down to room temperature, the homogenized pregrown culture (5%, V/V) was inoculated into the flasks. The pulps were incubated at 28°C for up to 10 days in an oscillation incubator with shaking at 150 rpm. The samples inoculated with inactivated pregrown culture by sterilization were used as control. For each sample, three replicates were made.

Pulp slurry was sampled by sampling spoon at selected times (2, 4, 6, 8, 10 days). Flasks were shaken as far as possible to make the matrix evenly before sampling, and 2 g of pulp (oven dry weight) was taken out for each flask. The pulp slurry sampled were collected by centrifugation at 4°C at 8000 rpm for 10 min and then were washed twice in nylon cloth bags with 100 ml water for further experiments. The supernatants obtained by centrifugation were collected for the determination of enzymes activities.

### 2.5. Pulp Bleaching

Biotreated pulps and the control were adjusted to 10% consistency, with pH 3.0 in polyethylene bags, and firstly treated with 0.5% EDTA at 80°C in water bath for 30 min and then were bleached under the conditions of pulp consistency 10%, 80°C, NaOH 1.5%, Na_2_SiO_3_ 3%, MgSO_4_ 0.5%, and H_2_O_2_ 3% and pH of 10 to 11 for 2 h. After EDTA treatment and bleaching, the pulps were thoroughly washed to neutral with distilled water. The pH of pulp was adjusted by using H_2_SO_4_ 2 mol/L and NaOH 1 mol/L. After bleaching, the test sheets were prepared using the bleached pulps by a Buchner funnel containing a cloth, compressed between filter papers, and dried at room temperature (25–27°C) in a dark environment.

### 2.6. Pulp Yellowing

Four UVB-340 lamps (20 Watts, Xue Laite, China), which emit UV-Vis light with wavelength (*λ*) of 295–350 nm, and *λ*_max_ = 340 nm, were used for light-induced yellowing. The light intensities at the sample site were kept at 4.6 *μ*W/cm^2^, which were determined by an ultraviolet irradiation meter. Temperatures in the aging box were kept at about 27°C by a cooling fan equipped in the box to minimize the heating effect of the light source. After 24 h irradiation, samples were conditioned in dark room for 4 h before analysis.

### 2.7. Enzyme Assays

Xylanase activity was determined by mixing 1.5 mL (1%, w/v) of beech xylan solution prepared in 50 mM NaAc-HAc buffer of pH 4.8, and 0.5 mL of diluted enzyme, which was incubated at 50°C for 30 min. The reaction was stopped by adding 3 ml 3,5-dinitrosalicylic acid (DNS) reagent and boiled for 10 min [23]. *β*-xylosidase activity was determined in buffer (NaAc-HAc, pH 4.8, 50 mM) with 1 mM 4-Nitrophenyl beta-D-xylopyranoside (pNPX) and suitable amount enzyme (total volume of 1.0 mL) and incubated for 10 min at 45°C. The reaction was stopped by 0.5 mL of Na_2_CO_3_ (1M). The released p-nitrophenol (pNP) was determined at 420 nm [24]. Carboxymethyl-cellulase (CMCase) activity and filter paper activity (FPA) were measured using the DNS method according to the literature [25]. One unit of enzyme activity was defined as the amount of enzyme that liberates 1 *μ*mol of sugar (or pNP) equivalents per minute under the assay conditions.

Laccase activity was determined by determining the rate of oxidation of 2,2′-Azinobis-(3-ethylbenzthiazoline-6-sulphonate) (ABTS) [26]. LiP and MnP activities were determined according to the method described in the literature [27].

### 2.8. Pulp Brightness and Post Color Number

ISO brightness of pulp samples were determined by brightness color tester (YQ-Z-48A, Hangzhou light instrument development Co., Ltd., China). The degree of brightness reversion was expressed by post color number (PCN), which is calculated as follows:

(1) $$\begin{array}{c} \text{PCN}=100\left\{{\left[\frac{{\left(1-R_{\infty }\right)}^{2}}{2R_{\infty }}\right]}_{\text{after}}-{\left[\frac{{\left(1-R_{\infty }\right)}^{2}}{2R_{\infty }}\right]}_{\text{before}}\right\}, \end{array}$$

where *R* denotes ISO brightness of a thick pad at *λ* = 475 nm [28].

### 2.9. Ultraviolet-Visible Diffuse Reflectance (UV-Vis DR) Spectra

UV-Vis DR spectra of pulp samples were recorded on a UV-2550 spectrometer (Shimadzu, Japan) equipped with an ISR-2200 integrating sphere, with BaSO_4_ as a background reference. The reflectance spectra were converted to *K*/*S* values according to the Kubelka–Munk equation [29]. Difference UV-Vis absorption spectra (▽*K*/*S* vs. wavelength) were obtained by subtracting the reflectance spectrum (*K*/*S* vs. wavelength) of control sample (un-irradiated sheet from that of the aged sheet. The *K* and *S* are the absorption and scattering coefficients. When the hand-sheets are thicker than 30 g/m^2^, the change of S (scattering coefficient) value was very small, and it could be considered as constant [30]. In this case, *K*/*S* value was directly related to the amounts of chromophores and UV-active substances in the hand-sheet [5]. And ▽*K*/*S* was approximately linear to the changes in chromophores or UV-active substances, which can be assumed according to the Kubelka–Munk theory.

### 2.10. Chemical Composition Analysis

Chemical compositions of pulps include the contents of acid-soluble lignin (ASL), acid-insoluble lignin (AIL), cellulose, and hemicellulose, were measured according to National Renewable Energy Laboratory (NREL, USA) analytical methods [31]. Hexeneuronic acid (HexA) content in pulp was measured by UV-spectroscopy method described in the literature [32].

### 2.11. FTIR-ATR Difference Spectra

Fourier transform infrared-attenuated total reflectance (FTIR-ATR) spectra of pulps sheets were recorded with a Tensor 27 spectrophotometer (Brüker, Germany) [33]. The infrared difference spectra were obtained by spectra of pulp samples treated with *F. concolor* X4 minus that of samples without biotreatment (control).

### 2.12. Statistical Analysis

All of the FTIR-ATR spectra and UV-Vis DR spectra were representative of three independent experiments. The assays of pulp brightness, enzymes activities, and chemical components were performed in triplicate, and each experiment was repeated at least three times. The mean value and standard deviations were calculated using Microsoft Office 2010 Excell (Microsoft, USA). The differences were considered statistically significant at *P* < 0.05 determined by using *t*-Student one-tail test.

## 3. Results

### 3.1. Effects of *F. concolor* X4 Treatment on Pulp Brightness and Brightness Stability

**Figure 1:**
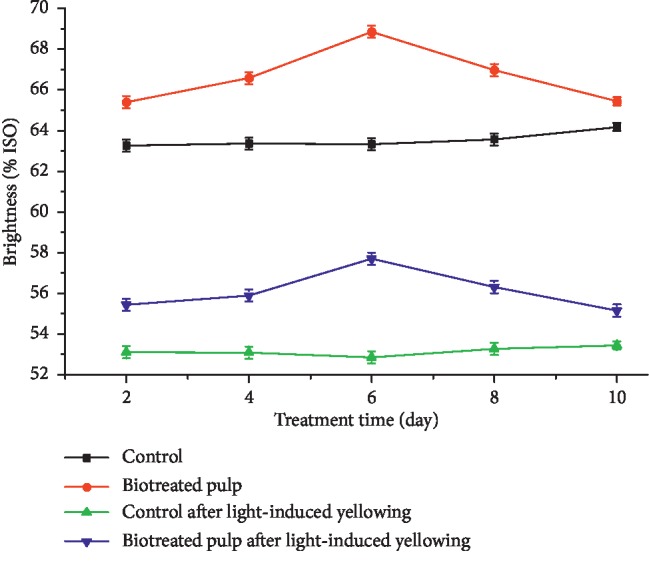
Effect of *F. concolor* X4 treatment on brightness of poplar CTMP before and after light-induced yellowing.

The pulp brightness of biotreated pulps and the control before and after light-induced yellowing are shown in Figure 1. It was found that the brightness of biotreated pulp samples was higher than that of the controls, either before or after light-induced yellowing, indicated that the *F. concolor* X4 treatment could improve pulp brightness. For example, the brightness of the biotreated pulp, respectively, increased by 5.0% and 12.2% (*P* < 0.01, *n* = 3) compared to the control after 2 and 6 days of *F. conco*lor X4 treatment before light-induced yellowing. Subsequently, the increase of biotreated pulp brightness decreased, and only 3.7% (*P* < 0.01, *n* = 3) increased compared to the control after 8 days of treatment.

After light-induced yellowing, the increases on pulp brightness were 5.9%, 11.4%, and 1.6% (*P* < 0.01, *n* = 3) for pulps obtained in 2nd day, 6th day, and 8th day treatment with *F. conco*lor X4 compared to the control, respectively.

**Figure 2:**
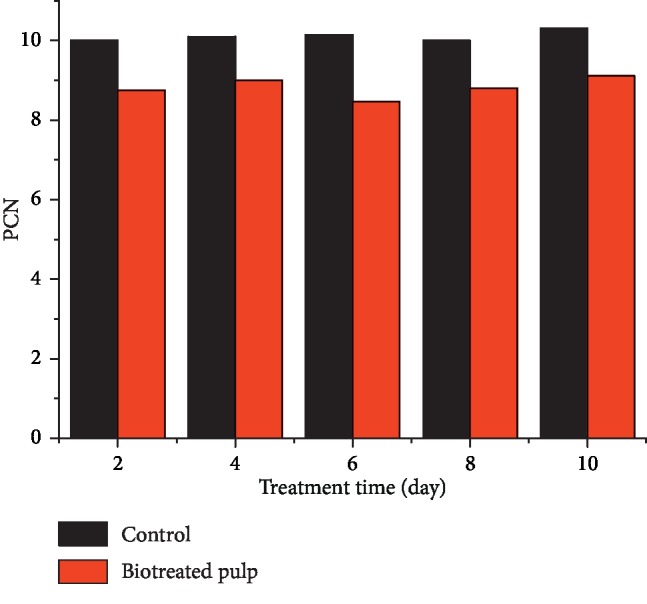
Changes of PCN of poplar CTMPs after treatment using *F. concolor* X4 for different times.

PCN is an index that expresses the pulps brightness reversion before and after yellowing.  Figure 2 shows the PCNs of poplar CTMPs biotreated for different times and controls after light-induced yellowing. Compared with the control, the PCNs of all the pulps treated with *F. concolor* X4 were decreased. For example, the PCN value decreased by 12.0% after 2 days of biotreatment compared to the control and by 10.9%, 15.8%, 12.0%, and 11.6% after treatment for 4 days, 6 days, 8 days, and 10 days, respectively.

During pulp treatment with *F. concolor* X4, samples obtained at different treatment time were centrifuged and different enzymes activities of the supernatant were detected (Table 1). It was found that LiP, xylanase, and cellulase appeared earlier, in which, the activities of LiP and xylanase reached 17.4 IU/L and 0.32 IU/mL (*P* < 0.05, *n* = 3) after treatment of 2 days. The MnP activity was 15.2 IU/L (*P* < 0.05, *n* = 3) at the 4th day of biotreatment. After 6 days treatment, laccase activity was detected (1 IU/L), and reached 1.2 IU/L at the 8th day. MnP and LiP activities peak appeared at the 10th and 8th day, and reached 29.8 IU/mL and 23.4 IU/L (*P* < 0.05, *n* = 3) respectively. The activities of CMCase and *β*-xylosidase were almost not detected during entry treatment period.

### 3.2. UV-Vis DR Spectra of Pulps

**Figure 3:**
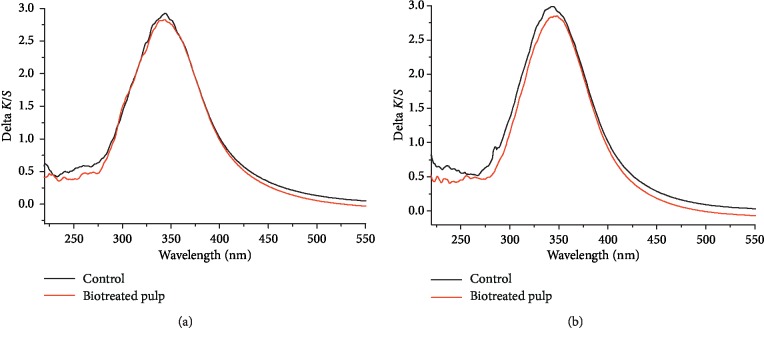
Difference UV-Vis absorption (▽*K*/*S*) spectra of poplar CTMP before and after treatment of 2 days (a) and 6 days (b), respectively, using *F. concolor* X4.

Figure 3 shows the UV-Vis DR spectra (▽*K*/*S*) of biotreated pulps and control before and after light-induced yellowing. The pronounced signal peaks appeared at around 340 nm. And the strengths of signals at around 240 nm and 280 nm for controls were higher than that of biotreated pulps. For the biotreated pulp, the ▽*K*/*S* values were lower than that of control, especially for the pulps obtained by biotreatment of 6 days (Figure 3(b)).

The chemical components of poplar CTMP before and after biotreatment of *F. concolor* X4 were determined and are listed in Table 2. It was found that compared to the control, the contents of lignin and hemicellulose of the biotreated pulps were decreased by 1.7% and 8.4% (*P* < 0.05, *n* = 4), respectively, after 2 days treatment of *F. concolor* X4, and by 3.1% and 12.6% (*P* < 0.05, *n* = 4), respectively, after 6 days of biotreatment. The HexA contents were also decreased by biotreatment. For example, decreased by about 6.8% (*P* < 0.05, *n* = 4) at the 2th day and 38.3% (*P* < 0.05, *n* = 4) at the 6th day, respectively.

### 3.3. FTIR-ATR Spectrum Analysis

**Figure 4:**
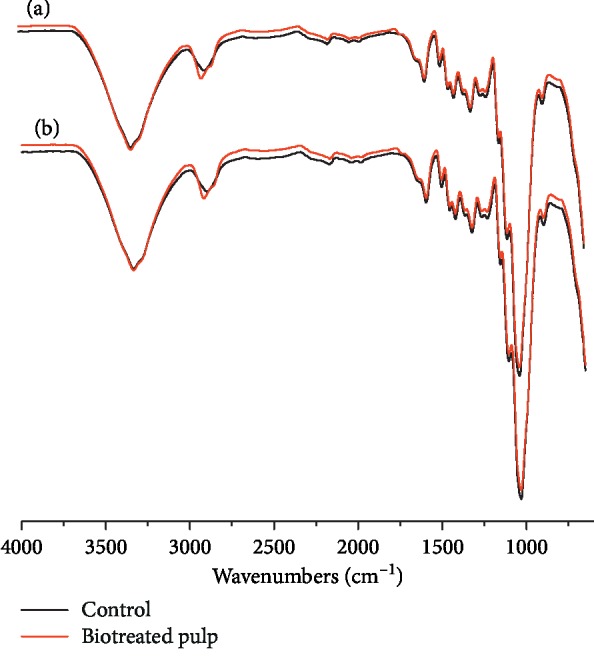
FTIR-ATR spectra of poplar CTMP before and after treatment of 2 days (a) and 6 days (b), respectively, using *F. concolor* X4.

FTIR-ATR spectra of poplar CTMP and control before and after treatment of 2 days and 6 days with *F. concolor* X4 are shown in Figure 4.  Table 3 shows some main absorption bands and their relative intensities [33–35].

It was indicated by the FTIR-ATR spectra that the intensities of certain bands were changed after biotreatment compared to the control. The absorptions at 896–897 cm^−1^ related to vibrations of anomeric carbon (*C*_1_) in hemicellulose, and the absorptions at 1160 cm^−1^, 1103–1108 cm^−1^, and 1052–1053 cm^−1^ which related to vibrations of C-O-C, O-H, and C-O bands, respectively, in cellulose and hemicellulose were decreased. Absorption at 1031 cm^−1^ related to hemicellulose or G type lignin vibrations and absorption at 1231 cm^−1^ related to C-O (syringyl) bending vibrations were also decreased compared to the control (Table 3).

## 4. Discussion

The different types of enzymes, include cellulase, xylanase, MnP, LiP, and laccase, could be produced during biotreatment of poplar CTMP with *F. concolor* X4 (Table 1). The enzymes could degrade hemicellulose, HexA, and lignin in the pulp, which was confirmed by analyzing changes in chemical components of the pulps before and after biotreatment (Table 2). It was known that hemicellulose, HexA, and lignin are light-sensitive and easy to be light-degraded to produce chromophores [36, 37], thus leading to light-induced yellowing of pulp. The brightness improvement of poplar CTMP treated with *F. concolor* X4 should be due to the degradation of hemicellulose, HexA, and lignin. The decrease in pulps brightness after 6 days of biotreatment may be due to the production of new chromophores from enzymatic degradation of lignin and repolymerization of degraded products of lignin [20]. For example, it has been reported that laccase has a role in the polymerization of lignin [38, 39].

Lower ▽*K*/*S* values (Figure 3) of biotreated pulps, compared to the control, indicate that biotreatment reduced the production of light absorbing substance (including chromophores and their precursors etc.). This is consistent with the changes in the pulp brightness (Figure 1) and PCN values (Figure 2). This may be mainly related to the degradation of lignin caused by lignin-degrading enzymes such as LiP, MnP, and laccase [40, 41] detected in pulp during biotreatment. The decrease of signal (▽K/S) at 240 nm and 280 nm after biotreatment (Figure 3) might be caused by the decrease of HexA content in biotreated pulp (Table 2). That HexA (240 nm) was easier to be degraded to the UV-active compounds (280 nm) and then transformed to colored structures during light-induced yellowing, which is consistent with the previous research results [42, 43]. *F. concolor* X4 treatment degraded HexA or changed HexA structures, thus blocking the transformation process of HexA to chromophores. Compared to the control, the decreases of ▽*K*/*S* around 340 nm for biotreated pulps (Figures 3(a) and 3(b)) suggested that less amounts of UV-active structures (aromatic carbonyl, etc.) could be generated after light-induced yellowing, which may from the degradation of aromatic-ring conjugated ethylenic bonds [44]. But the biotreatment using *F. concolor* X4 weakened the transformation process of these UV-active compounds (aromatic carbonyl, etc.) to chromophores (methoxyquinone, coniferyl aldehyde, o-quinone, etc.) during light-induced yellowing [5, 45], thus leading to high stability of pulp brightness.

In the FTIR-ATR spectra of pulps, relative intensities of absorption bands related to hemicellulose were reduced, indicating that hemicellulose in pulp was degraded after *F. concolor* X4 treatment, which was also proved by the decrease of hemicellulose contents shown in Table 2. Absorptions belonging to aromatic skeleton had no obvious changes except for syringyl lignin, suggesting that just limit degradation occurred after *F. concolor* X4 treatment and aromatic structure was not effectively destroyed. Table 2 also shows that the lignin content was just slightly decreased after biotreatment. These changes in hemicellulose and lignin were conducive to the brightness stability of pulps.

## 5. Conclusion

Biotreatment of poplar CTMP using *F. concolor* X4 increased the pulp brightness and inhibited light-induced yellowing of the pulp. It was found that different types of enzymes such as Lip, MnP, xylanase, laccase, a small amount of cellulase, etc. were produced in pulp during the *F. concolor* X4 treatment. The enzymes could degrade part light-active components such as lignin, hemicellulose, and HexA or change some UV-active structures in lignin, which may be some reasons for brightness improvement and the brightness stability enhancement.

## Figures and Tables

**Table 1:** The activities of enzymes produced by *F. concolor* X4 in poplar CTMP slurries during biotreatment.

Treatment time (day)	Enzyme activities						
	Lip (IU/L)	MnP (IU/L)	Laccase (IU/L)	CMCase (IU/ml)	FPA (IU/ml)	*β*-xylosidase (IU/ml)	Xylanase (IU/ml)
2	17.4 ± 2.4	Trace	Trace	Trace	0.01 ± 0.005	Trace	0.32 ± 0.05
4	19.6 ± 3.2	15.2 ± 3.1	Trace	Trace	0.01 ± 0.005	Trace	0.23 ± 0.05
6	21.0 ± 3.4	13.1 ± 2.4	1.0 ± 0.6	Trace	0.01 ± 0.005	Trace	0.23 ± 0.05
8	23.4 ± 2.8	10.5 ± 3.4	1.2 ± 0.4	Trace	0.01 ± 0.005	Trace	0.09 ± 0.05
10	20.1 ± 2.8	29.8 ± 3.1	0.6 ± 0.2	Trace	0.01 ± 0.005	Trace	0.05 ± 0.01

Data represent mean ± SD, *n* = 3.

**Table 2:** Chemical components of poplar CTMP before and after treatment using *F. concolor* X4 at different treatment times.

Pulps		Lignin (%)	Cellulose (%)	Hemicellulose (%)	HexA (*μ* mol/g)	Others (%)
2 day	Control	35.5 ± 0.6	44.5 ± 0.3	14.3 ± 0.4	4.4 ± 0.4	5.7
	Biotreated pulp	34.9 ± 0.3	46.1 ± 0.2	13.1 ± 0.2	4.1 ± 0.3	5.9

6 day	Control	35.1 ± 0.3	44.1 ± 0.3	14.3 ± 0.1	4.7 ± 0.4	6.5
	Biotreated pulp	34.0 ± 0.3	46.9 ± 0.2	12.5 ± 0.2	2.9 ± 0.4	6.6

Data represent mean ± SD, *n* = 3.

**Table 3:** Signal assignment in FTIR-ATR spectra of poplar CTMP and changes in spectra intensity before and after treatment of 2 days and 6 days using *F. concolor* X4.

Wavenumber (cm^−1^)	Band assignment	Relative intensity^a^			
		2 days		6 days	
		Control	Sample	Control	Sample
3333–3336	O-H (–OH groups) stretching vibrations	1.37	1.50	1.37	1.53
2896–2899	C-H (-CH_2_-,-CH_3_) stretching vibrations	0.62	0.64	0.62	0.65
1593	Aromatic skeletal stretching vibrations	0.81	0.84	0.82	0.87
1503	Aromatic skeletal stretching vibrations	0.70	0.73	0.71	0.73
1459	C-H- deformation; -CH_3_ and -CH_2_- asymmetric vibrations	0.86	0.89	0.89	0.90
1422–1429	Aromatic skeletal vibrations	1	1	1	1
1315–1317	CH_2_ vibrations in cellulose and hemicellulose	1.09	1.08	1.10	1.06
1204–1231	C-O (syringyl) bending vibrations	1.06	0.99	1.03	0.96
1160	C-O-C stretching in cellulose and hemicellulose	1.61	1.54	1.51	1.44
1103–1108	O-H association band in cellulose and hemicellulose	2.61	2.58	2.60	2.53
1052-1053	C-O stretching vibrations in cellulose and hemicelluloses	5.22	4.97	5.20	4.86
1030-1031	Hemicellulose or G lignin vibrations	6.25	5.90	6.23	5.63
896-897	Anomeric carbon (C1) vibrations in hemicellulose	1.19	1.03	1.21	1.03

^a^Relative intensity was the ratio of the intensity of a given band to the intensity of the reference band at 1422–1429 cm^−1^.

## Data Availability

The data used to support the findings of this study are included within the article.
